# Prediction and identification of natural antisense transcripts and their small RNAs in soybean (*Glycine max*)

**DOI:** 10.1186/1471-2164-14-280

**Published:** 2013-04-24

**Authors:** Hu Zheng, Jiang Qiyan, Ni Zhiyong, Zhang Hui

**Affiliations:** 1 The National Key Facilities for Crop Genetic Resources and Improvement, Institute of Crop Sciences, Chinese Academy of Agricultural Sciences, Beijing 100081, China

## Abstract

**Background:**

Natural antisense transcripts (NATs) are a class of RNAs that contain a sequence complementary to other transcripts. NATs occur widely in eukaryotes and play critical roles in post-transcriptional regulation. Soybean NAT sequences are predicted in the PlantNATsDB, but detailed analyses of these NATs remain to be performed.

**Results:**

A total of 26,216 NATs, including 994 *cis*-NATs and 25,222 *trans*-NATs, were predicted in soybean. Each sense transcript had 1–177 antisense transcripts. We identified 21 trans-NATs using RT-PCR amplification. Additionally, we identified 179 *cis*-NATs and 6,629 *trans*-NATs that gave rise to small RNAs; these were enriched in the NAT overlapping region. The most abundant small RNAs were 21, 22, and 24 nt in length. The generation of small RNAs was biased to one stand of the NATs, and the degradation of NATs was biased. High-throughput sequencing of the degradome allowed for the global identification of NAT small interfering RNAs (nat-siRNAs) targets. 446 target genes for 165 of these nat-siRNAs were identified. The nat-siRNA target could be one transcript of a given NAT, or from other gene transcripts. We identified five NAT transcripts containing a hairpin structure that is characteristic of pre-miRNA. We identified a total of 86 microRNA (miRNA) targets that had antisense transcripts in soybean.

**Conclusions:**

We globally identified nat-siRNAs, and the targets of nat-siRNAs in soybean. It is likely that the *cis*-NATs, *trans*-NATs, nat-siRNAs, miRNAs, and miRNA targets form complex regulatory networks.

## Background

Small RNAs play a crucial role in the regulation of gene expression in eukaryotes [1-5]. They are known to be involved in various aspects of genome stability, development, and response to biotic and abiotic stress [6]. Small RNAs regulate gene expression by modulating mRNA degradation, translational repression, and chromatin modification [1-6]. According to their origin or function in plants, these small RNAs are classified as microRNAs (miRNAs), natural antisense transcript (NAT) small interfering RNAs (nat-siRNAs), *trans*-acting short interfering RNAs, heterochromatic siRNAs, and long small interfering RNAs (lsiRNAs) [7-10].

NATs are a class of endogenous RNAs that have sequences partially, or completely, complementary to each other [11]. Based on their origin, NATs can be classified as either *cis* or *trans*. *cis*-NATs are formed from sense and antisense transcript that is transcribed from the same genomic loci, whereas *trans*-NATs have sense and antisense transcripts derived from different genomic loci [12-16]. NATs form double-stranded RNA (dsRNA) molecules with complementary sequences, and these dsRNAs are processed by Dicer-like proteins to generate nat-siRNAs [9]. These nat-siRNAs can be incorporated into the RNA-induced silencing complex (RISC) and act to guide the cleavage of complementary transcripts [9,17]. A transcript may form more than one trans-NAT with multiple antisense transcripts. These antisense transcripts can also form a trans-NAT with other transcripts. This process demonstrates the complexity of NAT involvement in the regulatory networks at the post-transcriptional level [15]. NATs are involved in numerous biological processes in plants. The expression of NAT genes can be tissue-specific, and many NATs are formed in response to environmental stimuli [11,15,18]. Several nat-siRNAs play roles in salt stress, bacterial resistance, cell wall biosynthesis, and fertilization in plants [9,17,19,20].

NATs are widespread in plant cells. In rice (*Oryza sativa*), 23.8% of genes exhibit antisense expression [21]. In *Arabidopsis*, more than 30% of the genome produces transcripts from both strands, and 25% of genes have antisense expression [22]. In bread wheat (*Triticum aestivum*), serial analysis of gene expression using tags revealed that 25.7% of unique genes exhibit antisense transcription [23]. Based on full-length cDNA and genomic data, 1,340 *cis*-NATs and 1,320 *trans*-NATs were predicted and identified in *Arabidopsis*[11,24]. In rice, 344 *cis*-NATs and 7,142 *trans*-NATs were identified to be formed by protein-coding genes [15]. The use of high-throughput sequencing data for small RNAs allowed the construction of a plant NAT database (PlantNATsDB) containing approximately two million NATs from 69 different plant species [25]. NATs and other small RNAs are annotated in the PlantNATsDB based on Gene Ontology categories (http://www.geneontology.org/). A total of 46,367 genes in the PlantNATsDB were used to predict 436 *cis*-NATs and 77,903 *trans*-NATs in soybean (*Glycine max*). However, the details for the soybean NATs remain to be determined.

Here, we report the prediction of 994 *cis*-NATs and 25,222 *trans*-NATs based on 66,213 soybean transcripts downloaded from the Phytozome database (version 1.0; http://www.phytozome.net/index.php) [26]. A total of 21 trans-NATs were identified by RT-PCR amplification. In all, 189,348 small RNAs, 27,465 of which were unique, were derived from 6808 NATs. These small RNAs were found to be enriched in the overlapping regions of NATs. The use of deep sequencing of the degradome is broadly applicable for global identification of small RNA targets [27-30]. Analyses of the soybean degradome database [31,32] identified 446 genes as the targets of 165 nat-siRNAs in soybean. Furthermore, we detected five *trans*-NAT transcripts that can be folded into the stem-loop structures that are characteristic of pre-miRNAs, and identified 86 targets of soybean miRNA that contained antisense transcripts in soybean.

## Results and discussion

### Prediction of NATs in soybean

**Figure 1:**
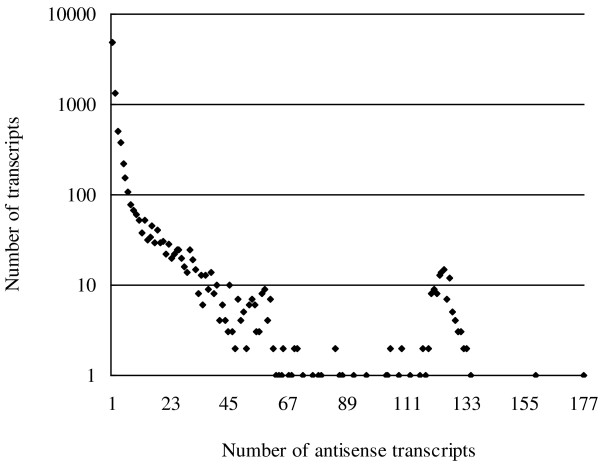
**Distribution of antisense transcripts in soybean.** For the 26,216 NATs, the antisense transcripts were sorted according to gene serial number and the number of repeats counted for every transcript. Transcripts had a range of 1–177 antisense transcripts in soybean.

We analyzed 66,213 soybean transcripts downloaded from the Phytozome database (http://www.phytozome.net/index.php) [26]. Over 13% (8,634) of the transcripts had at least one antisense transcript in soybean. Among these transcripts, over 50% (4,788) had only one antisense transcript, while the others had from 2 to 177 antisense transcripts (Figure 1). A total of 26,216 NATs were identified in soybean. The NATs were categorized into *cis*-NATs and *trans*-NATs according to the transcript origin from the genomic loci. Mapping of the NAT transcripts to the soybean genome identified 994 *cis*-NATs and 25,222 *trans*-NATs (Additional files 1 and 2).

### *cis*-NATs and *trans*-NATs in soybean

The *cis*-NATs can be classified into three types: convergent (with 3’-ends overlapping); divergent (with 5’-ends overlapping); and enclosed (with one transcript completely overlapping the other) [15]. Among the 994 soybean *cis*-NATs, 468 were arranged in the enclosed orientation; 291 were convergent; and 235 were divergent (Additional file 1). In contrast, most of the *cis*-NATs from *Arabidopsis* and rice are convergent [11,15].

**Figure 2:**
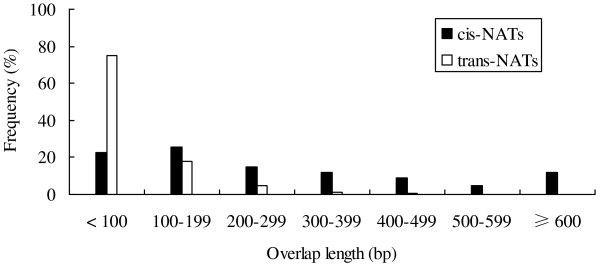
**Length distribution in the overlapping regions of *cis*-NATs (black) and *trans*-NATs (white).**

*cis*-NAT overlaps length are usually longer than *trans*-NAT overlaps length [14], and this was also true for soybean NATs. The *cis*-NAT overlaps length ranged from 31–2,808 bp (308 bp average), whereas the *trans*-NAT overlaps length ranged from 31–1,716 bp (87 bp average). The overlapping length of the majority of *trans*-NATs (74.87%) was shorter than 100 bp, and only 7.31% were longer than 200 bp (Figure 2).

Many transcripts have multiple antisense transcripts in plant. For the *cis*-NATs, several genes are involved in two *cis*-NATs in *Arabidopsis*[11]. In soybean, we identified 11 transcripts that formed two or more *cis*-NATs with other transcripts (Table 1). *Glyma13g11820.1* and *Glyma13g11940.1* had ten and three antisense transcripts respectively. The large genomic sequence sizes of *Glyma13g11820.1* (78,178 bp) and *Glyma13g11940.1* (101,408 bp) may help to explain the reason they contained multiple antisense transcripts.

**Table 1:** **Genes forming *cis*-NATs with multiple antisense transcripts**

**Gene 1**	**Gene 2**	**Overlap length (bp)**	^**1**^**Type**
*Glyma02g14410.1*	*Glyma02g14420.1*	216	enclosed
	*Glyma02g14430.1*	333	convergent
*Glyma02g36840.1*	*Glyma02g36830.1*	149	divergent
	*Glyma02g36850.1*	53	convergent
*Glyma04g34480.1*	*Glyma04g34490.1*	62	enclosed
	*Glyma04g34500.1*	113	convergent
*Glyma05g22920.1*	*Glyma05g22910.1*	118	divergent
	*Glyma05g22930.1*	63	enclosed
*Glyma08g01140.1*	*Glyma08g01150.1*	69	enclosed
	*Glyma08g01160.1*	1932	convergent
*Glyma13g11820.1*	*Glyma13g11830.1*	428	divergent
	*Glyma13g11840.1*	593	enclosed
	*Glyma13g11850.1*	428	enclosed
	*Glyma13g11860.1*	230	enclosed
	*Glyma13g11870.1*	192	enclosed
	*Glyma13g11880.1*	186	enclosed
	*Glyma13g11890.1*	162	enclosed
	*Glyma13g11900.1*	271	enclosed
	*Glyma13g11910.1*	118	enclosed
	*Glyma13g11920.1*	72	enclosed
*Glyma13g11940.1*	*Glyma13g11950.1*	147	enclosed
	*Glyma13g11960.1*	118	enclosed
	*Glyma13g11970.1*	542	enclosed
*Glyma15g37560.1*	*Glyma15g37570.1*	69	enclosed
	*Glyma15g37580.1*	45	divergent
*Glyma17g31520.1*	*Glyma17g31510.1*	137	convergent
	*Glyma17g31530.1*	118	divergent
*Glyma17g36410.1*	*Glyma17g36400.1*	677	enclosed
	*Glyma17g36420.1*	304	divergent
*Glyma18g03060.1*	*Glyma18g03050.1*	58	convergent
	*Glyma18g03070.1*	453	divergent

^1^The types of *cis*-NAT were categorized as enclosed (full overlap), convergent (3’-ends overlap), or divergent (5’-ends overlap).

**Figure 3:**
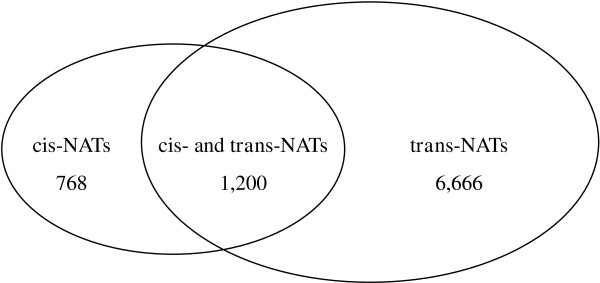
**Distribution of transcripts in the NATs.** All 8,634 NAT transcripts were categorized as *cis*- or *trans*-NATs. Of these, 768 transcripts formed only *cis*-NATs, 1,200 transcripts formed both *cis*- and *trans*-NATs, and the other 6,666 transcripts were *trans*-NAT transcripts.

For the *trans*-NATs, one transcript commonly had many antisense transcripts [15,24]. The number of antisense transcripts ranged from 1 to 177 in soybean, possibly a consequence of the homologous genes in the gene families frequently having the same antisense transcript [24]. The soybean genome has gone through at least two rounds of polyploidy and subsequent diploidization events. Segmental duplications and chromosome-level homology are common in the soybean genome [33-36], and approximately 75% of genes have multiple copies [37]. Some transcripts can form both *cis*-NATs and *trans*-NATs [15]. Of the 8,634 transcripts in soybean, 1,200 transcripts were involved in both *cis*- and *trans*-NATs (Figure 3). These genes may be regulated by *cis*- and/or *trans*-NATs.

### Identification of NATs in soybean

We identified 17 transcripts using RT-PCR amplification. These 17 transcripts can form 21 trans-NATs. One transcript may form NATs with multiple antisense transcripts [15]. We identified *Glyma01g09920.1*, *Glyma04g05850.1* and *Glyma08g42710.1* as having the same five antisense transcripts. The overlapping region in the sense transcripts had similar sequences (Additional file 3). *Glyma14g13230.1* can form NATs with *Glyma02g34100.1*, *Glyma10g23170.1*, *Glyma14g22790.1*, *Glyma18g16420.1*, *Glyma14g22790.1* and *Glyma20g06230*.1. The overlapping region of *Glyma02g34100.1*, *Glyma10g23170.1*, *Glyma18g16420.1* and *Glyma14g22790.1* had the same sequences, while *Glyma20g06230*.1 can form NAT at another location on the *Glyma14g13230.1* transcript.

### Small RNAs originating from NATs

**Figure 4:**
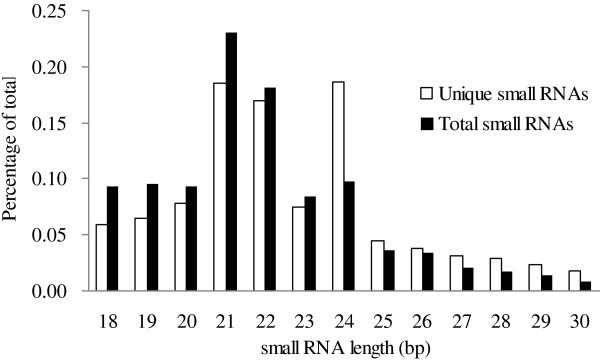
Size distribution of unique (white) and total (black) small RNAs derived from NATs.

As NATs can generate small RNAs [14,15] we searched for the presence of small RNAs in our library for the 8,634 transcripts. We identified 2,286 transcripts able to give rise to small RNAs. 189,348 small RNA sequences, representing 27,465 unique small RNAs, were generated from these transcripts. The most abundant unique small RNAs were 21, 22, and 24 nt in length (Figure 4). In soybean, these 2,286 transcripts could form 179 *cis*-NATs and 6,629 *trans*-NATs (6,808 total; Additional file 4).

**Figure 5:**
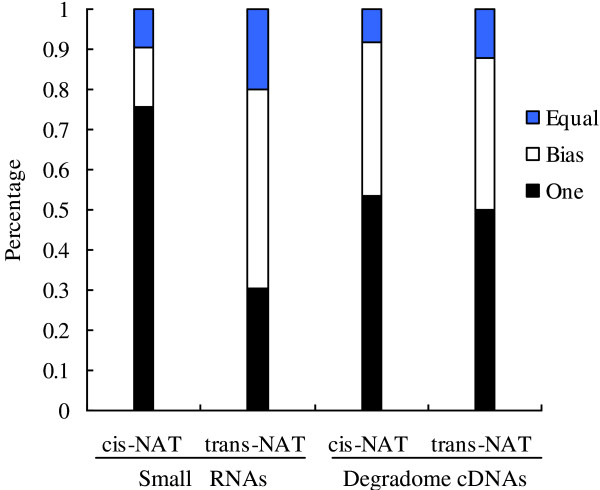
**Distribution of small RNAs and degradome cDNAs on the NATs.** 6,808 NATs (179 *cis*-NATs and 6,629 *trans*-NATs) small RNAs were generated in our study. Small RNAs and NAT associated degradome cDNAs were counted. The ratio of sense and antisense transcripts was calculated as follows: One (only one transcript of NATs generated small RNAs or degradome cDNAs); Equal (0.5 ≤ ratio ≤ 2); and Bias (ratio < 0.5 or > 2).

Most of the small RNAs were derived from one of the NAT transcripts in *Arabidopsis*[15]. Both *cis*- and *trans*-NATs mostly generated small RNAs from one strand of the NAT in soybean (Figure 5). Among the *cis*-NATs, 75.4% (135) generated small RNAs from only one strand of the NAT, and 9.5% (17) generated small RNAs equally from both transcripts. For the *trans*-NATs, 30.4% (2,019) generated small RNAs from only one strand, and 19.9% (1,321) generated small RNA equally from both strands.

Small RNAs originated from both the overlapping and non-overlapping regions of NATs [15]. The distribution of small RNAs in these two regions varies in different plants [38]. In soybean, the average densities (the number of small RNA loci per kilobase) of the unique and total small RNAs in the overlapping regions were 103.84 and 517.80, respectively, and 48.72 and 344.24 for the entire NATs. T-tests for the unique (*P* < 0.0001) and total (*P* < 0.0001) small RNAs suggested that both were enriched in the overlapping region.

### The NATs degradome in soybean

NATs can produce small RNAs, which suggests that these transcripts are excised by Dicer-like proteins. We searched for the degradome tags of the 6,808 NATs that could produce small RNAs. A total of 122 *cis*-NAT and 4,425 *trans*-NAT transcripts were identified as having degradomes (Additional file 4). Most degradome tags were derived from one NAT transcript (Figure 5): 53.3% (65) *cis*-NATs, and 50.2% (2,222) *trans*-NATs, generated tags from only one transcript. This was consistent with the small RNA bias towards one strand of NATs.

### Identification of NAT-derived small RNA targets in soybean

nat-siRNA can regulate gene expression by guiding target mRNA degradation at the posttranscriptional level [9,19]. The targets of siRNAs can be globally identified by analyzing the degradome [27-32]. We searched the nat-siRNA targets by analyzing the soybean degradome and identified 446 target genes for the 165 nat-siRNAs (Additional file 5). Of these 165 nat-siRNAs, 83 were derived from *trans*-NATs, 81 from *cis*- or *trans*-NATs, and only one was generated from a *cis*-NAT. Regarding the 446 target genes, 203 were targeted by a nat-siRNA derived from the corresponding NAT sense strand, and 75 were targeted by a nat-siRNA produced from the corresponding antisense strand. The nat-siRNAs targets not only the transcript of their own NATs but also that of other transcripts. A total of 168 genes were identified as targets of nat-siRNAs, these nat-siRNAs were not produced from target sense or antisense transcripts.

### miRNAs may be involved in the formation of NATs in soybean

Some NATs can form stem-loop structures and generate mature miRNAs. In rice, some miRNAs are derived from the overlapping transcript antisense of MADS box transcripts, and act to guide MADS transcript cleavage [39]. We used the UNAfold program to simulate folding of 2,286 transcripts identified as being able to produce small RNAs [40]. Five transcripts were predicted to contain a stem-loop structure characteristic of pre-miRNA (Additional file 6). These transcripts were *Glyma02g02440.1*, *Glyma04g38430.1*, *Glyma05g03670.1*, *Glyma05g32980.1*, and *Glyma05g37200.1*. Further analysis revealed that *Glyma04g38430.1* and *Glyma05g32980.1* were miR166 genes; *Glyma05g37200.1* produced miR319; and *Glyma02g02440.1* and *Glyma05g03670.1* generated small RNAs randomly from both sense and antisense strands (Additional file 7). These five genes may be involved in the biogenesis of both miRNAs and NATs. There are two possible pathways by which miRNAs could be generated from these transcripts. One pathway occurs when the sense and antisense transcripts are co-expressed in the same cell, form a double RNA duplex, and produce nat-siRNAs. This then guides the generation of small RNAs from their sense or antisense transcripts [9]. Another pathway occurs when the sense and antisense transcripts are not co-expressed in the same cell; these transcripts can fold into a hairpin and produce miRNAs.

Targets of miRNAs may be involved in the formation of NATs. We collected 596 candidate targets of miRNAs and searched for targets that could form NATs. 86 miRNA targets were identified as having antisense transcripts (Additional file 8). These targets could form *cis*- and *trans*-NATs. Analysis of the soybean degradome of these 86 targets validated 28 as being miRNA targets [31,32].

### NATs may form complex regulatory networks in soybean

It has been suggested that NATs form complex regulatory networks in plants [15]. One transcript often has many antisense transcripts, and these can form NATs with other transcripts. In soybean, 1,200 transcripts were predicted to form both *cis*- and *trans*-NATs (Figure 3). 11 transcripts had multiple *cis*-NATs. Soybean commonly has one transcript that has many antisense transcripts forming the *trans*-NATs. Of the 8,634 transcripts that form NATs, 3,846 contain multiple (2–177) antisense transcripts (Figure 1).

The nat-siRNAs play important roles in plant development. NATs produce nat-siRNAs via a process mediated by Dicer-like RNA-dependent RNA polymerase and Suppressor of Gene Silencing 3. The nat-siRNA is then incorporated into the RISC and directs the cleavage of a complementary mRNA [9,17]. With high-throughput sequences of small RNAs from different soybean tissues, we detected 6,808 NATs that produced at least one small RNA (Additional file 4). These small RNAs potentially regulate gene expression at the posttranscriptional level. In recent years, deep sequencing of the degradome has been used extensively to globally identify small RNA targets. Analysis of the soybean degradome database enabled identification of 446 genes as targets of 165 nat-siRNAs. These nat-siRNAs targets included NAT sense or antisense transcripts, and other transcripts (Additional file 5).

miRNAs and their targets may be involved in NAT regulatory networks. Five transcripts with pre-miRNA stem-loop structures had antisense transcripts. These transcripts may generate nat-siRNAs or miRNAs; this is dependent on whether the transcripts are co-expressed with antisense transcripts in the same cell. Furthermore, we detected 86 miRNA targets that had antisense transcripts in soybean. These miRNA targets might be regulated by their antisense transcripts.

**Figure 6:**
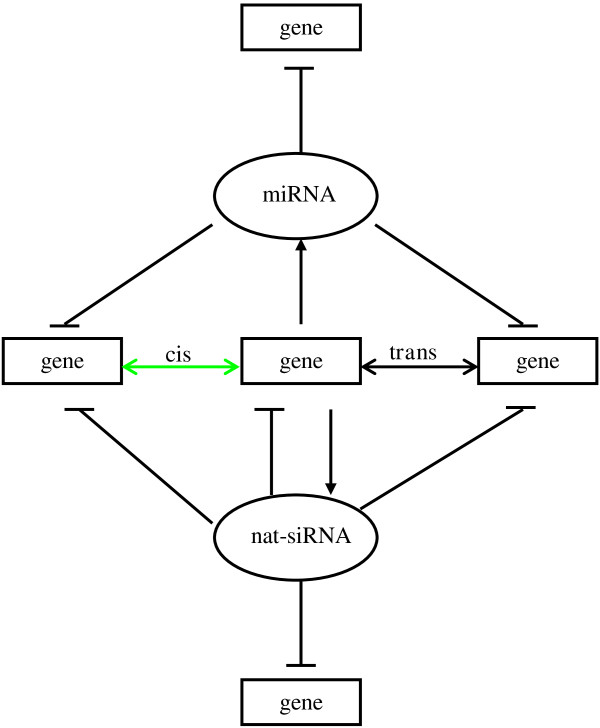
**The complex regulatory networks of NATs.** In the NAT regulatory networks genes may form *cis*- and *trans*-NATs. Some NATs may fold into the hairpin structure characteristic of pre-miRNAs, and generate miRNAs; some NATs may give rise to nat-siRNAs. The nat-siRNAs can self-regulate the expression of NAT sense or antisense transcripts, and they can target other genes. Additionally, many miRNA targets may be involved in the formation of NATs.

NATs may form complex regulatory networks in soybean (Figure 6). In these networks, gene expression is regulated by other genes forming *cis*- or *trans*-NATs. NATs can produce nat-siRNAs that self-target their NAT transcripts and other gene transcripts. Some NATs produce miRNAs to regulate expression of other genes, and some miRNAs guide the cleavage of NATs.

## Conclusions

We globally predicted NATs in soybean and confirmed the identity of 21 trans-NATs by RT-PCR. The use of high-throughput sequencing of the small RNAs and degradome in soybean enabled the identification of 27,465 unique NAT-derived small RNAs, and 446 targets of 165 nat-siRNAs. The identification of these nat-siRNA targets can help to determine the function of nat-siRNAs in soybean. Furthermore, we identified five pre-miRNAs, and 86 miRNA targets that contained antisense transcripts. NATs, NAT-derived small RNAs, nat-siRNA targets, NAT-related pre-miRNAs, and NAT-related miRNA targets, may form complex regulatory networks. It follows that an understanding of these networks will further our understanding of the roles that NATs play in soybean development.

## Methods

### Plant material and RNA isolation

Soybean (Glycine max) seeds of the cultivar Williams82 were planted in the experimental station of the Institute of Crop Sciences at the Chinese Academy of Agricultural Sciences, in Beijing in May. Flowers were collected and quickly frozen in liquid nitrogen and then stored at −70°C for use in future RNA isolation. Leaves and roots were collected from 12 days old soybean seedlings. Total RNA from different tissues was isolated separately using TRIzol reagent (Invitrogen, Carlsbad, CA, USA) according to the manufacturer’s instructions. RNA samples were evaluated by electrophoresis on a 1% agarose gel.

### Sequence datasets

Soybean gene sequences and annotations were downloaded from the Phytozome database (version 1.0; http://www.phytozome.net/index.php) [26]. The small RNAs and the degradome were previously identified with deep sequencing in our laboratory. Information for the soybean small RNAs and degradome is from the NCBI-GEO database (accession no. GSE33380). The soybean miRNAs were downloaded from miRBase (version Release 18; http://microrna.org/) [41].

### Prediction of NATs in soybean

NATs were detected by aligning predicted *Glycine max* cDNA sequences to each other. If a pair of overlapping genes were matched at opposite strands with an E-value ≤ 1e-9^19^, then they were defined as a NAT pair. The NAT pair was located on the soybean genome to identify *cis*- and *trans*-NATs. If a pair of NATs was located at the same genome locus, they were considered a *cis*-NAT pair. If they were located at different genomic loci, they were considered a *trans*-NAT pair. Based on the overlap between the two transcripts, the *cis*-NATs were categorized into three types: convergent (3’-ends overlap); divergent (5’-ends overlap); and enclosed (full overlap).

### Identification of NATs by RT-PCR

Several NATs were identified by use of RT-PCR. We designed gene-specific primers to amplify cDNAs based on their NAT transcript sequence (Additional file 9). 50 μg leaf RNA, 25 μg root RNA, and 25 μg flower RNA were added to a tube and mixed gently, these RNAs were treated with DNase I (Fermentas, Harrington, Ontario, Canada) for 30 minutes at 37°C, and then purified with phenol-chloroform. A total of 4 μg purified RNA was used in a 20 μl RT reaction containing 2 μl gene-specific RT primer (10 μM) (Additional file 9), 4 μl 5× reaction buffer, 1 μl RiboLock RNase inhibitor (20 u/μl), 2 μl dNTP (10 mM), and 1 μl (200 u/μl) RevertAid M-MuLV reverse transcriptase; this was carried out using the RevertAid First Strand cDNA Synthesis kit (Fermentas, Harrington, Ontario, Canada) according to the manufacturer’s instructions. 1 μl of the first strand cDNA sample was used as template for subsequent PCR reactions in 25 μl reactions using gene-specific primers with the following cycle conditions: 95°C, 30 s; 55°C, 30 s; 72°C, 1 min; the run was for 35 cycles. The RT-PCR products were evaluated by electrophoresis on 2% agarose gel. 1 μl of RT-PCR product was ligated into the pGEM-T vector using the pGEM-T easy vector system (Progema, Madison, WI, USA) according to the manufacturer’s instructions; next, 2 μl ligation reaction was transformed into TOP10 competent cells. Five clones of each gene were sequenced (Additional file 10).

### Analysis of small RNAs

The small RNAs were screened against the Sanger Non-coding RNA Database (http://www.sanger.ac.uk/resources/databases/rfam.html) to eliminate rRNAs, tRNAs, and snoRNAs [42]. Small RNAs that were identical to the transposable elements identified in the *G. max* genome, downloaded from SoyTE (http://www.soybase.org/soytedb/), were also removed. Small RNAs were aligned to NAT transcripts using SOAP [43]. Sequences that were identical to NAT transcripts were considered as NAT-derived siRNAs. The significance of the enrichment of small RNAs in the overlapping regions of NATs was calculated according to the method previously described by Chen et al. [25]. Briefly, the number of unique small RNAs generated from the overlapping region (No) and NAT transcripts (Nt), and their corresponding lengths (Lo and Lt) were determined. The ratios No/Nt and Lo/Lt were used to calculate the density of small RNAs in the overlapping (Do) and entire (Dt) regions of the NAT. The ratio of Do/Dt was considered to be the enrichment score, and a standard χ^2^ test was performed to test the significance of the enrichment of small RNAs in the overlapping regions of NATs.

### Identification of nat-siRNA targets

Degradomes were mapped onto NAT transcripts using SOAP. The sequences that identically matched NAT transcripts were considered to be the NAT degradome. The locus sequence, containing the 20 bp upstream and downstream regions of the NAT degradome, was extracted as the long degradome from the transcript. Next, a search for small RNA targets was performed as described by Schwab et al. [44]. Total NAT-derived small RNAs were used to query the long degradome sequences, and small RNAs and complementary cDNA pairs for potential target sites were obtained using Patscan set at the default parameters: three mismatches, zero insertions, and zero deletions were permitted [45]. Only hits with fewer than two mismatches in positions 1–9, no mismatches in positions 10 and 11, and fewer than three mismatches after position 11 in the small RNAs were considered good target sequences.

### Identification of pre-miRNAs and miRNA targets involved in the NAT network

Flanking sequences of the small RNAs that matched identically with the NAT transcripts were obtained as described by Sunkar and Zhu [46]. Fragment sequences 200 bp upstream and downstream of NAT-derived small RNAs were extracted from the NAT transcripts. Simulation of folding was then performed using UNAfold [40]. Identified secondary structures were checked for miRNA features using MirCheck [47]. The miRNA targets in soybean were predicted using the psRNATarget server (http://plantgrn.noble.org/psRNATarget/) [48]. Validated targets of miRNAs in soybean were obtained from Song et al. and Hu et al. [31,32]. The targets containing the antisense transcripts were considered to be NAT-related miRNA targets.

## Competing interest

The authors declare that they have no competing interests.

## Authors’ contributions

ZH drafted the initial manuscript. ZH and QJ performed the bioinformatics analysis. QJ performed the small RNA library construction. ZN carried out the tissue collection, RNA extraction and RT-PCR. HZ contributed to the design of the study, obtained the funding, and finalized the manuscript. All authors read and approved the final manuscript.

## Supplementary Material

Additional file 1 **The *cis*-NATs in soybean.** The overlap length and types of *cis*-NATs are shown. Click here for file

Additional file 2 **The *trans*-NATs in soybean.** The overlap length is shown. Click here for file

Additional file 3 **Identification of NATs in soybean.** The transcripts were amplified by RT-PCR and sequenced. The overlapping regions are shown at the location of the transcript sequence. Click here for file

Additional file 4 **The small RNAs and degradome cDNAs of NATs.** Unique and total small RNAs and unique and total degradome cDNAs are shown. Click here for file

Additional file 5 **Identification of nat-siRNA targets in soybean.** The degradome, their abundance, and the genes from which they were derived are shown. nat-siRNAs, nat-siRNA target sites, nat-siRNA abundance, and the origin of the nat-siRNAs are indicated. nat-siRNAs can target the sense or antisense transcript of a given NAT and other transcripts. The origins of nat-siRNAs are shown as a, s, and o. a: the nat-siRNA derived from the antisense transcript of a given NAT targets the sense transcript; s: the nat-siRNA derived from the sense transcript of a given NAT targets the sense transcript; o: the nat-siRNA derived from a given NAT guides the expression of another gene. Searches for genes that generate the nat-siRNAs identified nat-siRNAs derived from different genes. Nat-siRNAs that identically matched more than five sense or antisense transcripts are denoted as multiple. Click here for file

Additional file 6 **Secondary structures of five NAT-related pre-miRNAs.** Five NAT transcripts had the stem-loop structure that is characteristic of pre-miRNAs. Of these five transcripts, two were miR166 pre-miRNA and one was miR319 pre-miRNA. The miRNAs are indicated in red. Click here for file

Additional file 7 **The small RNAs were matched to the five NAT-related pre-miRNAs.** The small RNAs are shown along with information on their length, abundance, and location in the pre-miRNAs. Click here for file

Additional file 8 **The miRNA targets containing antisense transcripts.** The miRNA targets could form *cis*- and *trans*-NATs with other genes. a: These targets were validated previously by the analysis of the degradome in soybean [31,32]. Click here for file

Additional file 9 RT-PCR primers used for the amplification of NATs. Click here for file

Additional file 10 The sequences of transcripts identified by RT-PCR. Click here for file
