# Supplementary material for: Prediction and identification of natural antisense transcripts and their small RNAs in soybean (Glycine max)
Source: BMC Genomics. 2013 Apr 24;14:280. doi: 10.1186/1471-2164-14-280 (PMC3643859; doi:10.1186/1471-2164-14-280)
Supplement: Additional file 6 — Secondary structures of five NAT-related pre-miRNAs. Five NAT transcripts had the stem-loop structure that is characteristic of pre-miRNAs. Of these five transcripts, two were miR166 pre-miRNA and one was miR319 pre-miRNA. The miRNAs are indicated in red. [file 1471-2164-14-280-S6.doc]

*Glyma02g02440.1*

A A A CUGCGUACA

CUCUUUGC UAUGC AGGGUA GG A

GAGAAGCG AUACG UUCCAU CC C

- C A CCCUCCCUA

*Glyma04g38430.1* (pre-miR166)

UU - C AA

UUGAGGGGAAUG GG CUGG UCGAGGCUUU--UC A

AACUCCCCUUAC UC GACC GGCUUCGGAA AG G

U- G A \/ GA

~20bp

*Glyma05g03670.1*

- C -| A GGGAGGGA UG

GG CUCUU GC UAUGCGA GGUAUGG UGU \

UC GAGAA CG AUACGUU CCAUGCC ACG U

G A U^ C G------- CA

*Glyma05g32980.1* (pre-miR166)

- UU ---| UU - CU UCACAAAGGA A GU

AGAUG GG GGUAU UGAGGGGAAUG GGC UGG CGAGGCUUU GGUUC CA \

UUUAC UC UCAUG ACUCCCCUUAC UCG ACC GCUUCGGAA UCAAG GU G

U CU AAA^ U- G AG UA-------- - AG

*Glyma05g37200.1* (pre-miR319)

A CUC U AG AG- G AC C AA AGCG

AAG GAGCUUUCUUCAGUCCA AUGGG GAC UAAGAUUCAAUU CU CCG UCAUUCAU CA UGUUGAGUGUA A

UUC CUCGAGGGAAGUCAGGU UGUCC UUG AUUCUAAGUUAA GA GGC AGUAAGUG GU ACGACUCAUAU A

C UCA U A- ACA G GU A AG AAAU
